# Investigating Moral Distress in Clinical Research Professionals—A Deep Dive into Troubled Waters

**DOI:** 10.1002/eahr.60006

**Published:** 2025-01-03

**Authors:** Elena Bosack, Dawn Bourne, Elizabeth Epstein, Mary Faith Marshall, Donna T. Chen

**Affiliations:** ^1^ Medical student at the University of Miami Miller School of Medicine; ^2^ Assistant professor at the University of Virginia School of Nursing and a clinical ethics consultant at the Center for Health Humanities and Ethics at the University of Virginia School of Medicine; ^3^ Professor of ethics and pharmacology at the University of Virginia School of Nursing and is on the core faculty at the University of Virginia Center for Health Humanities and Ethics; ^4^ Professor of public health sciences and a director of the Center for Health Humanities and Ethics at the University of Virginia School of Medicine; ^5^ Professor of health humanities and ethics, psychiatry, and public health sciences at the Center for Health Humanities and Ethics at the University of Virginia School of Medicine

**Keywords:** human subjects research, moral distress, hierarchy, research integrity, research ethics, racism, clinical research professionals (CRPs)

## Abstract

Moral distress occurs when professionals are constrained from taking what they believe to be ethically appropriate actions or are forced to take actions they believe are ethically inappropriate, challenging their professional identities and representing systems‐level issues within organizations. Moral distress has been recognized in a variety of health care‐related fields; however, the phenomenon is still comparatively unexplored among clinical research professionals (CRPs). In this qualitative study, we interviewed ten CRPs to unearth root causes of moral distress in this ethically unique profession. Four themes emerged from the data as contributors to moral distress: commodification of research; concern for research participants; compromised science; and structures of hierarchy. The experience of racism as a source of moral distress is also explored. The findings of this study indicate that the existence of moral distress in clinical research is troubling not only for the welfare of CRPs but also for the greater clinical research enterprise.

While great progress has been made in protecting research subjects and advising researchers on ethical practices, the moral complexities of clinical research remain an important ethical challenge—particularly for those on the front lines, such as clinical research professionals. “Clinical research professional” (CRP) is an umbrella term that encompasses a variety of titles, backgrounds, and roles. The Society of Clinical Research Associates lists 15 duties of a CRP that include both administrative tasks like protocol design and development of informed consent documents, as well as interactive tasks like recruitment and enrollment of research participants and protection of participants’ rights.^1^ In general, CRPs work under the authority of a principal investigator (PI) and are bound to conduct studies only as outlined by the study protocol and under the surveillance of institutional review boards (IRBs). Most CRPs work directly with study participants; they may feel substantial anguish if, despite following protocol, they feel they are not doing all they can to protect their research participants. Regardless of their intentions, making decisions that stray from study protocol can result in study termination and job loss. Considering these constraints to decision‐making, it is reasonable to suspect that clinical research professionals experience moral distress.

The concept of moral distress describes situations where professionals are “constrained from taking what they believe to be ethically appropriate actions or are forced to take actions they believe are ethically inappropriate” and consequently “feel complicit in acting unethically and are unable to fulfill important professional obligations.”^2^ While most scholarship surrounding moral distress has centered on nurses and other clinical care professionals, a modest collection of studies points to the existence of moral distress in the clinical research profession.^3^ These studies have paved the way for future research regarding moral distress in the field of clinical research. Our study aims to take a closer look at moral distress in the clinical research profession to gain a better understanding of its origins and some exacerbating factors, and to offer protective measures and potential solutions.

## STUDY METHODS

We designed this study as an interview‐based qualitative descriptive study with an initial goal of interviewing ten CRPs. Although effective tools have been developed to quantify moral distress in several health care professions, these tools were created using established causes of moral distress.^4^ Thus, the applicability of these quantitative tools to novel populations such as CRPs is uncertain. Our study aims best align with the aims of qualitative descriptive research, which seeks to “capture all of the elements of an event that come together to make it the event that it is.”^5^ In contrast to quantitative research, qualitative descriptive studies allow more room for the unanticipated while remaining close to the data.^6^ Through open‐ended interviews, for example, participants can give form and meaning to their subjective experiences without the limitations imposed by predetermined scales. Allowing the participant to construct their own response in this way helps prevent the beliefs of the researcher from shaping the data to the researcher's preconceived notions.^7^ We used inductive coding with no predefined codes in a “bottom‐up” approach to extract data from interviews without introducing preconceived notions.^8^ We planned to use the process of coding and theme identification from the initial set of interviews to assess for data saturation and to guide the need for further interviews.

### Recruitment and interview process

A recruitment email was sent to several CRP organizational listservs across multiple institutions and a convenience sample of ten respondents were scheduled for an interview. All participants met the inclusion criteria of having at least six months of experience as a CRP in the past five years. Each participant (hereafter “interviewee” for clarity) was provided with the definition of moral distress in the recruitment materials and self‐identified as having experienced moral distress in their role as a CRP. At the start of their interview, all interviewees were reminded of the definition of moral distress. Throughout the process, we paid special attention to avoid conflating moral distress with similar but discrete concepts such as emotional distress and ethical dilemmas in order to avoid improper characterization of moral distress.^9^ IRB approval for this study was granted by the University of Virginia IRB‐SBS under protocol #4728.

The interviewer (EB) conducted one‐on‐one semistructured Zoom interviews with each interviewee to discuss their experiences. Interviews ranged in length

**Our study aims to take a closer look at moral distress in the clinical research profession to gain a better understanding of its origins and some exacerbating factors, and to offer protective measures and potential solutions**.

from 30‐95 minutes, with an average length of 59.5 minutes. The automatic transcripts generated by Zoom for each interview were edited and verified by EB using an audio recording of the meeting, and any potentially identifying information was redacted.

### Coding and theme identification

The primary coders (EB and DB) read and independently coded all transcripts. Inductive codes centered around descriptions of conflicts that caused moral distress and the interviewee's response to each conflict. After a first pass, the coders developed a shared coding vocabulary. Using this code vocabulary, each transcript was then re‐coded with additional codes and retroactively applied as needed.

After each transcript was coded, the primary coders met via Zoom to cross‐check codes and validate code reliability. Disagreements were typically resolved in discussion between the primary coders, but on two occasions a third coder (EE) was asked to independently code the discrepant section.

During these discussions, the coders made a point of verifying that the anecdotes being described in the transcript met the definition of moral distress. Those anecdotes that did not meet the definition were not coded or included in the analysis. One transcript was excluded in its entirety. A second was flagged for potential exclusion and, after several discussions amongst all the authors, was retained and discussed separately as an emergent concept in moral distress.

After all transcripts were coded, codes were revisited and refined. Codes that were used in only one transcript were evaluated for analytical similarity to other codes and were merged, with one exception that will be discussed in detail. Codes were then categorized conceptually into themes and subthemes. All authors read the transcripts and participated in identification of themes and subthemes through discussion of preliminary categories identified by the coders with the goal of achieving consensus on the themes and subthemes. During theme identification, the authors returned to the transcripts to ensure emerging themes were grounded in the words of the interviewees.

### Ensuring data trustworthiness

The rigor of our coding was strengthened by intra‐ and inter‐rater reliability. The primary coders (EB and DB) coded each transcript at least twice and codes were confirmed between each pass. Coded transcripts were shared between both coders and assessed repeatedly, with a third coder (EE) on call for additional input. Through the theme development process, the authors were able to achieve consensus on the identified themes and subthemes. The authors felt that thematic saturation was sufficiently robust at ten interviews after no new codes emerged in the final two interviews. In one case, member checking was performed to confirm that the interviewee's story was accurately represented.

## EMERGENT THEMES AND SUBTHEMES: ROOT CAUSES OF AND RESPONSES TO MORAL DISTRESS

The ten interviewees represented a diverse sample of CRPs: 20% of interviewees had six months to a year of experience, 40% of interviewees had 1‐9 years of experience, and 40% of interviewees had over ten years of experience as a CRP; 60% of interviewees had a background in patient care before becoming a CRP, and the majority (70%) did not hold a formal certification, which is typically not required to work as a CRP. Most interviewees (80%) worked in clinical trials, with interventions including medical device studies, drug trials, and novel treatments. The other 20% worked primarily in community‐based and population health‐focused clinical research. More precise details of study types were not obtained to maintain confidentiality. Table 1 contains demographic information as reported by interviewees during their interview.

**Table 1:** Interviewee Demographics

**Gender**			
Male		Female	
30%		70%	
**Years of CRP experience**			
6 months‐1 year	1‐4 years	5‐9 years	10+ years
20%	30%	10%	40%
**Patient care background**			
Yes		No	
60%		40%	
**Research setting**			
Industry	Academic		Both
30%	40%		30%
**Primary study type**			
Clinical trials/interventional		Public health/population health	
80%		20%	

Interviewees from diverse professional backgrounds conducting various types of research across multiple institutions all described situations of moral distress. The root causes of moral distress in clinical research professionals that emerged from these interviews were categorized into four themes: commodification of research; concern for research participants; compromised science; and structures of hierarchy (see table 2, available online; information about accessing this material is in the “Supporting Information” section at the end of this article). Interviewees’ responses to moral distress were also analyzed. One transcript described a situation of racial bias as a source of moral distress for the interviewee and was examined separately.

### Commodification of research

In the U.S. alone, the clinical trials market was valued at $57.76 billion in 2023 and is projected to increase steadily each year.^10^ Though our study was not limited to CRPs working in clinical trials, the most common theme emerging from the transcripts was the financial pressure felt by research professionals. Even in the absence of overt conflicts of interest—which are generally caught by IRBs—financial considerations exert an invisible but significant influence over decision‐making in clinical research. As one interviewee summarized, “Once you get an enrollment, you're paid a certain amount. Then, if you get the person to complete their follow‐up appointments, you get more money. And if they complete all the follow‐up appointments and finish out their time in the study, then you get additional money. And so some projects like this can be high dollar. And so when these [PIs] consider that, and they all know, roughly, how much money each enrollment will bring in, they're thinking big picture about the money. Which yes, it's important because the money is what's funding the research staff, but at the same time, morally, you need to be considering … how likely is this [research treatment] going to actually help the patient potentially versus kill the patient because the patient is too sick to handle the [research treatment]?” (I.6).

Many cases of moral distress resulting from financial considerations involved situations where the PI wanted to enroll an individual in a trial, but the CRP did not feel that study participation was right for the patient. In some instances, interviewees felt that some patients did not even qualify for the research study, so the inclusion and exclusion criteria were modified or “stretched” to enroll an individual. As one interviewee explained, “I didn't feel like anyone was ever fully coerced into it, but a lot of times, I felt like sometimes, maybe, due to whatever reasons, a patient maybe didn't quite want to be in the study or didn't quite hit all the [inclusion/exclusion criteria]—it felt like they [the PI] kind of stretched things a little bit to kind of meet all those competing demands” (I.2).

These subtle manipulations not only had an impact on the integrity of the research being conducted (as we will discuss later) but also on the CRP's self‐image as a research professional. According to another CRP, “One part of clinical research is a little bit like sales, which I didn't know. You do have to sort of convince people to do it because that's how I get paid. My position isn't funded by my institution at all; it's only paid by the numbers of people I get to enroll. So if I don't enroll, I'm not going to make money. So that right there is a conflict, but I think that's how most positions are funded here. If your grant runs out of money or doesn't meet its endpoint or something like that, you would not have a job. They're all funded like that, that I know of, at least. And so, I think the sales part of it is a little bit hard” (I.1).

This sales metaphor echoes language quoted in a 2018 study of clinical research nurses who complained of “feeling like reluctant salespeople” when recruiting study participants.^11^ This salesperson role introduces a new and undesired identity that competes with CRPs’ internal values and motivations and compromises the quality of research delivery while producing a significant emotional burden.^12^ Our study shows that moral distress occurs when CRPs must balance their financial duty to enroll participants and their moral duty to care for them.

### Concern for research participants

Many interviewees described situations of moral distress where their concern for study participants complicated their duties as a CRP. While this theme manifested in many ways, one of the most common was where the CRP felt that study enrollment was not in the best interest of the patient—or potential participant. One CRP elaborated on this theme: “Sometimes we meet patients that are just really sick … sometimes we just look at the patient or look at their chart review and think ‘They are really sick and maybe palliative care is probably the best option for them.’ But sometimes our PI sort of wants to push them to try a clinical research trial—I mean, of course, they don't force them to—but they really try to advocate for it. And sometimes we just don't agree on that judgment call. In our [the CRP team's] minds, we're like ‘I think they're ready to just enjoy the life that they have without us intervening and getting them to do all these studies and these tests and exams and all of that.’ So sometimes there's disagreement between what I might feel is appropriate and best for the patient and what my PI is suggesting. And, ultimately, because I work for the PI … we can't just blatantly say ‘No, I don't agree with you’” (I.10).

Stories like these were common among interviewees in our study and are common threads in the tapestry of moral distress. Indeed, the term moral distress was first coined in 1984 by Jameton, a bioethicist, to describe his nursing students’ reactions to providing treatments that they considered harmful or overly burdensome.^13^ Since then, across the literature and across clinical professions, moral distress has been consistently linked to potentially inappropriate care.^14^

At the same time, the ethical demands of clinical research are distinct from those of clinical care. In clinical research, one is expected to respect the needs of participants while also understanding that, unlike in clinical care, the primary goal may not involve healing the patient. Rather than “benefiting” individual participants per se, clinical researchers are tasked with the “protection” of human subjects while at the same time balancing a broader moral responsibility to advance scientific knowledge for the benefit of society.

This conflict was also characterized in a 2019 qualitative study of clinical research nurses, who described a feeling of dual obligations regarding their identity as a nurse versus their identity as a *research* nurse.^15^ Interviewees expressed the difficulty of navigating the complex interface “between advancing science for the greater good and caring for the individual [research subject] in the best possible way.”^16^ Although many interviewees in our study did not have a background in nursing, this concept of dual obligations was still an obvious source of moral distress. This interplay underscores the multifaceted nature of moral obligations within the realm of clinical research, where the commitment to both individual participants and to the greater societal good coexists.

Such friction between clinical research and clinical care was expanded beyond a binary in an earlier study of ethical challenges among study coordinators, where Davis et al. identified three contrasting duties of a CRP: patient advocacy, subject advocacy, and study advocacy.^17^ As they describe, “there are times when one advocacy must advance and the others retreat, and deciding which ones to focus upon and which ones to subrogate is the major ethical challenge of the study coordinator position.”^18^ While CRPs are responsible for the conduct and integrity of a study, they must balance this responsibility with not only the protection of study participants, but also the best interests of patients considering study participation. In a notable parallel to our cohort, the study coordinators in Davis et al.'s study also singled out improper enrollment of patients: “Enrollment … is the [principal investigator's] priority, but that's not necessarily [the CRP's] priority.”^19^

Other sources of moral distress invoking CRPs’ concern for study participants included situations where the CRP felt that the boundaries between research and clinical care had become blurred, and the participant did not understand how their rights as a study participant differed from their rights as a patient. In many cases, this would lead the CRP to wonder whether the informed consent given by the participant was truly “informed.” As one interviewee noted, “Like when they know that the physician who is running the research study is also involved in their care for after, I'd sometimes feel that there may have been pressure for them to want to try to please the provider, because they know that they're involved in it in both ways, if that makes sense. I wasn't really sure, sometimes, if they were saying yes [to the study] because they fully wanted to participate [in the study], or if they were saying yes because they wanted to improve the outcome of what they were going to have done [in the clinic] and also improve the relationship that they were having with that provider” (I.2).

This blurring of boundaries represents a moral conflict that is unique to clinical research as opposed to clinical care.^20^ Interestingly, in our study, the blurring of boundaries did not only appear to cause moral distress in and of itself but also pushed CRPs to question the nature of informed consent given by study participants. Without proper informed consent, the ethical foundation of clinical research becomes compromised, potentially eroding trust between researchers and participants and undermining the integrity of the entire research endeavor.

### Compromised science

Regarding CRPs’ twofold obligations to study participants and to the study itself, CRPs also expressed concern over the integrity of the research being conducted, even in cases where it posed no harm to the participants. As mentioned above, multiple interviewees pointed to the perceived subjectivity of the inclusion and exclusion criteria, which were often described as being “stretched” to “fit” participants into studies. As one CRP summarized, “If you're going to keep making that bar a little bit lower at each point, then at which point does it become wrong to just keep stretching things like that” (I.2).

Another interviewee expressed concern over possible data manipulation that, although not harmful to study participants, conflicted with their perceived duties as a CRP. They explained that “[t]here were times that [the PI] did something wrong, [they] would contact [the electronic record program] and have them delete it. Or, [the PI] would go in, fill this out, and then write a note: ‘This was completed during the visit.’ Well if it was completed during the visit, you had time to do it in your tablet right then. So that was a huge issue. It just wasn't appropriate. And definitely I don't believe that this was life‐threatening or anything like that. But it definitely wasn't following the protocol guidelines that we had of the things that we needed to do at each visit, and it was my responsibility to make sure that these things were done” (I.3).

As implied above, CRPs also experienced moral distress when they felt they could not rely upon their coworkers or PI to ethically fulfill their professional duties. Another interviewee described this type of situation: “You can learn a lot, if not more, from a bad investigator than a good investigator. Because you're doing preventative—you're covering all bases, you're making sure things aren't missed. The patients had to come in once every [x] weeks, and [the PI] had to do a full physical exam—and that is listening to your heart, lungs—and then neuro[logical] exam—having them walk, do the eye exam, all these things. [The PI] was so fast at it. One time, I actually timed [the PI], and it was a minute. [They] did quote unquote ‘the whole thing’ in a minute. I was like, ‘This is wrong!’” (I.5).

The inability to trust coworkers to perform their responsibilities correctly is a common source of moral distress as it can generate feelings of powerlessness or create additional responsibilities for the CRP as well as foster a distrustful and uncooperative work environment.^21^ This issue was often raised together with a lack of reliable study oversight from independent third parties, who may have caught and corrected issues before they progressed to causing moral distress. Similarly, many interviewees complained of insufficient training, in both their own experience and that of their coworkers, which perpetuated moral distress‐inducing mistakes. As their role as a CRP involves upholding the scientific integrity of a study, CRPs who lack appropriate training are essentially set up for professional and ethical failure.

### Structures of hierarchy

Moral distress is especially prominent in situations where ethical decision‐making occurs within a tight hierarchical structure. In clinical research, the PI is often in charge of the practical execution of the study, and the CRP works under the PI's authority. Study leadership may also extend above the PI to include sponsoring companies, organizational or institutional leadership, or academic administration. As noted in Davis et al.'s “The Invisible Hand in Clinical Research,” despite its many important and unique roles, the position of CRP is often seen as an assistant‐level position—if it is seen at all.^22^ This hierarchy can give rise to moral hazard, where morally important decisions are made by an authority who is not implicated in the consequences of their own decisions. In other words, the decision‐*makers* are not the decision‐*bearers*.^23^ Indeed, many interviewees described study leadership's lack of receptivity to problems that the CRP considered morally distressing. This feeling is clearly described in the following comment of one interviewee: “I guess the only other thing would just be when you have a PI that's not receptive to feedback and gets angry, that's a real big barrier. And I think that can make it really hard to voice concerns too. Our PI, [they] were just not wanting to hear any advice, and I think that was really hard for everybody that was working on the big study. It's just like, ‘ugh,’ we can't voice anything to this person. That was really tough” (I.4).

Due to the hierarchical structure of the research team, those who detected and were affected by the problems were unable to effect solutions. One CRP described the leaders of a study attempting to recruit participants at the beginning of the Covid‐19 pandemic against the advice of both the CRPs tasked with recruitment and the intended participants themselves: “They [the study leaders] weren't listening to the people on the ground … you're forcing me to ignore what the people on the ground are saying. And they [the ‘people on the ground,’ i.e., the study population] are all saying ‘What are you doing with this research; why are you doing this now?’” (I.4).

Previous studies in similar populations have shown that hierarchy and organizational culture have an enormous influence on the moral well‐being of research staff.^24^ In a study of community‐based researchers conducting drug‐use research, for example, Fisher et al. found that researchers who perceived their organizations to be committed to high ethical standards experienced lower levels of what they termed “work‐related moral stress.”^25^ This recognition in the moral distress literature has warranted a conceptual shift, moving beyond individual conflicts to acknowledge moral distress as a systemic issue.^26^ Within this framework, the locus of conflict extends beyond an individual's internal value dynamics to instead highlight organizations and systems that prevent the prioritization of moral imperatives.

To make matters worse, CRPs in our study who detected problems were often prevented from acting by fear of retribution. Two interviewees described situations where they had spoken out to study leadership about morally distressing situations but were either ignored or punished. This in turn amplified the CRPs’ fear of retribution, as they worried they would be held responsible if higher authorities cracked down on the study over which they ostensibly had little to no control. One interviewee described how they tried to alert the monitoring agency about their PI cutting corners: “There were things [on the study record] that I was like, ‘Review these things. Look at these things.’ But also, you're in a spot where you can't put your job in jeopardy. I couldn't afford to get fired …We all needed our jobs. Needed to pay my mortgage. Needed to feed my dog. Needed to feed myself. And that just put us in a really bad position of always being on edge. Not knowing what would happen that would come back on us, that we did something [wrong] when we knew that we didn't” (I.3).

It is crucial to acknowledge that these morally distressing situations are not simple communication problems or professional disputes. CRPs expressed profound dismay at finding themselves complicit in research practices they perceived as ethically unsound, yet found themselves bereft of agency to effect change. This situation is not tenable from a moral standpoint, neither for the CRPs nor for the research participants involved.

### Responses to moral distress

Given these aspects of powerlessness and fear of retribution, it should come as no surprise that one of the most common responses to moral distress among our interviewees was self‐mitigation. This quiet acquiescence and assumption of additional responsibilities is expressed clearly in the following quote: “You just know at a certain point no one's going to do anything, even if you go barking off. So you just have to do what you have to do to make sure things are covered. So from that moment on, I decided I would do—as best I could as a [CRP]—my own exam of the patient before [the PI] did. I would really go over ‘Are you having any problems? Is there any vision problems?’ I would do more questions; I wasn't listening to their heart and lungs. But I wanted to make sure [the PI] wasn't missing something even though [they] were signing off saying [they] did the full physical exam …. So that helped me to be more assertive without being aggressive, because [the PI] would not have taken that well” (I.5).

While self‐mitigation may be temporarily effective, it is ultimately unsustainable. Expecting those afflicted with moral distress to be responsible for overcoming their moral distress increases the burden on the individual and ignores the fact that moral distress reflects a systems‐level issue.^27^ It follows that quitting—either quitting one's current position or leaving the clinical research profession entirely—proved another frequent response to moral distress among our interviewees. Other passive responses included deferring to higher‐ups on the study (“But if [the PI] told me to do something, I would defer to [them]. I would just do what [they] say, and just absorb whatever negative feeling I might have” [I.10]) and avoidance (“So there were sometimes ways where we could avoid certain doctors or things, but that wasn't always the case. … I tried to do my best to work around some of those things. It was stressful” [I.6]). These passive responses do nothing to address or alleviate moral distress, and as shown by the excerpts, ultimately worsen the situation by compromising the CRP's moral integrity and creating additional stress.

In contrast, two responses had the potential to reduce moral distress. In some cases, CRPs mentioned coming together with their coworkers in solidarity, which in turn empowered them to address the moral distress head‐on. While this may be a cooperative subtype of self‐mitigation, its utility in empowering CRPs should distinguish it as a potential strategy to mitigate moral distress in clinical research.

Once empowered, CRPs in our study were often able to confront their moral distress by speaking out. It should be noted that in most cases, speaking out alleviated moral distress by either (1) alerting their PI of their distress and moving toward a solution or (2) alerting oversight bodies of distressing situations and encouraging them to invoke their authority. In two cases, however, the moral distress was worsened by speaking out, as the CRP was ignored (I.4) or faced retribution from the PI (I.3).

Positive responses to moral distress can and should serve as inspiration to provide mechanisms that institutions can use to alleviate or prevent moral distress in their employees. By encouraging solidarity among CRPs and empowering CRPs to have a voice, research organizations may be able to foster a more cooperative and cohesive workplace that produces high‐quality research. One such mechanism that has demonstrated positive utility is consultation services, which provide a protected space for parties to share concerns without fear of retribution. As one interviewee reasoned: “I know in the clinic side of hospitals have—for like doctors and nurses—they have the ethical consult teams, and there may have been something at the institution I was at for research, but not that I'm aware of at least. But if there was something like that, where if it was a kind of tricky gray‐zone area, something like that, you could call instead of someone directly tied up to your department, because I feel like it's kind of hard to get an unbiased view if you called your manager or the provider carrying out the study saying ‘I don't think this patient is quite understanding.’ But I feel like somebody who's distanced or detached from the situation itself who you could maybe call up and have them mediate the discussion between you and the other people involved could be something useful” (I.2).

In recent years, some institutions have organized Research Ethics Consultation Services (RECS) to do just this. Distinct from administrative bodies like IRBs, the roles of RECS include “assisting with research design and implementation, providing a forum for deliberative

**The origins of moral distress within the CRP population are multifaceted, revealing both significant parallels and distinctions when compared to studies on moral distress within clinical care populations**.

exploration of ethical issues, and supplementing regulatory oversight.”^28^ In other words, RECS provide a more organized avenue for researchers to access expert ethical advice, work through ethical issues, and negotiate courses of action.^29^ Though RECS are still in their nascence, they could meet a crucial need to prevent and mitigate moral distress in CRPs. Some institutions have also created Moral Distress Consultation Services to specifically address moral distress in health care workers;^30^ institutions with these services could expand them to include CRPs.

The following excerpt demonstrates some of the causes and effects of CRPs’ responses to moral distress. In this quote, the interviewee is responding to the interviewer's question, “When you think about the causes of these events, how do you think this could be changed?”: “I think one thing that can help influence change is for people like [CRPs] who are working under an authority figure like a PI, for us to just have the courage to speak up when we feel that maybe something is questionable or it's not in the best interest of the patient. I think hierarchy makes us feel like we're not able to speak up, but if the [CRPs] working under PIs just were willing and had the courage to do so and did not feel like we were doing something wrong by speaking up, I think that might change the overall culture of how we approach this. Other than that, I'm not quite sure” (I.10).

Here, the interviewee expresses an internalized sense of responsibility for their own moral distress—perhaps the ultimate example of self‐mitigation. It is our hope as authors that, rather than place the onus of responsibility on the CRPs suffering moral distress, we can work to identify institutional fixes that do not force CRPs to muster the courage to speak up, but rather empower and reward them for doing so.

## A PREVIOUSLY UNACKNOWLEDGED SOURCE OF MORAL DISTRESS

While other interviewees encountered moral distress within the parameters of the classical definition of the term, one interviewee discussed an experience they attributed to a source previously undescribed in the moral distress literature: racism. Although this theme was only raised by one interviewee, the research team felt that it was too important to exclude.

When asked about experiences of moral distress, one interviewee described a situation where they felt that due to their ethnicity and accent, they had been judged unfairly and treated in a hurtful manner by a patient being recruited to participate in a study. After multiple attempts to accommodate the patient, the interviewee was ultimately compelled to act against their conscience. In describing the situation during the interview, the CRP said: “I told my PI that [the patient was] declining participating in the research. However, I'm a hundred percent sure if a native speaker had talked to [the patient], she would give them the consent. It's all about me. So that's why I don't like this situation, and I feel like this is very distressful, very offensive. I didn't tell the truth, but I [did what I] had to, you know? It's something annoying and something that hurts my feelings, [if I were to] tell my PI [that] I couldn't get a consent from somebody because I'm not a native speaker. So, I just said [the patient] declined, but I did not tell anybody about this situation … because it hurt me” (I.8).

The interviewee did not tell their PI the whole truth, an action inconsistent with what they believed was the “right thing to do.” It should be noted that, unlike in many of the situations described earlier in this paper, this interviewee had a good relationship with their PI. According to the interviewee: “My PI [is] super nice and [is] a very understanding [person] … But in this particular situation, I did not tell anybody …When you are angry, you don't make good decisions. I felt so sad and I didn't want to talk about it, just like it never happened” (I.8). Thus, even the support of an authority figure was not enough to mitigate the frustration and shame of discrimination, and the interviewee was forced to take an action that they perceived to be inappropriate to protect their professional and personal dignity. As they explained: “And this is the part that I feel sad about, that I had to lie and I had to say [the patient] declined. But if I had told the truth, or had told the story as is—one of my colleagues [is] a native speaker, so [they] would had gotten consent from [the patient]—but I felt, at that time, if I did that, that would hurt my feelings more, because—in this world, I feel that we are all the same. So, whenever I feel that I couldn't do something and my colleague [could do] it just because where I was born and raised, this is offensive. I felt that way. I might be wrong, but I felt that way. If my colleague could [have gotten] consent from [the patient], why [couldn't] I?” (I.8).

The language used here is telling, as the interviewee repeatedly describes doing what they “had to” do in contrast to what they felt they should have done. Racism is not a new concept in systems‐level ethical analyses, yet it remains unexplored in the moral distress domain. As of this writing, no studies have systematically evaluated the convergence of actual or perceived racism and moral distress, although one Canadian guide reasoned that “lived experiences of racism and discrimination can collide with the moral stress of working in healthcare and put racialized healthcare workers at an increased risk of experiencing moral distress.”^31^

And while it has not been previously evaluated within the context of moral distress, according to the definition of moral distress—i.e., occurring when professionals “are constrained from taking what they believe to be ethically appropriate actions or are forced to take actions they believe are ethically inappropriate” and as a result “feel complicit in acting unethically and are unable to fulfill important professional obligations”^32^—we found no justification within this framework to exclude the experience described by the interviewee as moral distress triggered by racism.

Interestingly, this case differed from those discussed above in that both speaking out and finding solidarity with coworkers would have worsened rather than alleviated the interviewee's moral distress. Whereas other interviewees found solace in these responses, in this instance, the interviewee expressed that deferring to a native‐speaking CRP or recounting the situation would have been “offensive” and would have served only to “hurt [the interviewee's] feelings more.” As such, the interviewee was compelled to take an action they believed to be ethically inappropriate and consequently felt unable to fulfill their professional obligations and roles—consistent with the definition of moral distress. It is worth exploring whether the creation of safe spaces for CRPs to candidly discuss these types of experiences might help mitigate moral distress as the research enterprise seeks to be more inclusive of racially and ethnically diverse people among research professionals and research participants.^33^

## STUDY LIMITATIONS AND FUTURE DIRECTIONS

This study cannot be used to indicate the severity or prevalence of moral distress in this population as our sample is not statistically representative of all CRPs and no attempt was made to quantify the levels of moral distress expressed by participants. To quantify these aspects, future researchers may consider using data presented by this study to develop an instrument to measure moral distress in CRPs similar to the Moral Distress Thermometer or the Measure of Moral Distress for Healthcare Professionals (MMD‐HP) used in other populations.^34^ Additionally, it should be noted that our interviewees self‐selected for interviews and so may represent a subset of individuals who either experience moral distress at abnormally high levels, motivating them to participate, or abnormally low levels, enabling them to recall experiences that others might find too difficult to discuss.

Despite these limitations, this study achieved data saturation from ten interviewees with a wide range of experiences. We were able to catalog and assess both standard and novel features of moral distress in a population that has been neglected in the literature. Nevertheless, given the relatively small sample and the ever‐evolving nature of the clinical research environment, additional qualitative studies could uncover emerging developments or interrogate a particular research arena more intensively. We hope that future researchers will build off the foundational data assembled here to further address moral distress in the clinical research profession.

## CONCLUSION

This study has shown that moral distress is experienced by CRPs in a variety of domains. The origins of moral distress within the CRP population are multifaceted, revealing both significant parallels and distinctions when compared to studies on moral distress within clinical care populations. This study should serve as a call for institutions to begin addressing moral distress among CRPs. Additionally, the repeated emergence of financial issues as a root cause of moral distress should prompt critical reflection on the economic organization of the clinical research enterprise.

Since one interviewee felt that racism was a root cause of their moral distress, we wonder why the existing moral distress literature does not address racial discrimination as a source. It could be argued that conflating moral distress with racism could confuse efforts to mitigate either. In any case, a thorough treatment of racism in the clinical research profession is beyond the scope of this paper, although future researchers should consider the role racism may play in moral distress.

The implications of moral distress in clinical research are far‐reaching. The nature of research as a field provides space for moral distress to reverberate in a variety of domains. While no egregious scientific blunders were reported as a result of moral distress, subtle errors could, if widespread, unsettle the entire field of research. Thus, sources of moral distress in CRPs should be promptly recognized and addressed, both for the sake of CRPs and for the very ideal of scientific advancement.

## ACKNOWLEDGMENTS

The interviews were conducted as part of Elena Bosack's undergraduate senior thesis while a student at the University of Virginia. Funding for this study was provided by an Ingrassia Family Echols Scholars Research Grant. Donna Chen was the Bioethics Lead of the integrated Translational Health Research Institute of Virginia (iTHRIV) and was supported in part by the National Center For Advancing Translational Sciences of the National Institutes of Health under Award Number UL1TR003015; iTHRIV also provided in‐kind support and guidance to the research team. The content is solely the responsibility of the authors and does not necessarily represent the official views of the National Institutes of Health. The research team would like to thank each of our interviewees who volunteered their time to speak about their experiences. Moral distress is not an easy thing to discuss, and we salute each interviewee's candor and thank them for their confidence.

## Supporting information

Table 2 is available in the “Supporting Information” section for the online version of this article and via *Ethics & Human Research*'s “Supporting Information” page: https://www.thehastingscenter.org/supporting-information-ehr/.

Supporting information

## References

[1] “Definition of a Clinical Research Professional,” Society of Clinical Research Associates, accessed January 19, 2022, https://www.socra.org/certification/certification-program/definition-of-a-clinical-research-professional.

[2] Epstein, E. G. , et al., “Moral Distress, Mattering, and Secondary Traumatic Stress in Provider Burnout: A Call for Moral Community,” AACN Advanced Critical Care 31, no. 2 (2020): 146–57, at 149. 32525997 10.4037/aacnacc2020285

[3] Fisher, C. B. , et al., “Moral Stress, Moral Practice, and Ethical Climate in Community-Based Drug-Use Research: Views from the Front Line,” AJOB Primary Research 4, no. 3 (2013): 27–38; Sunderland, N., et al., “Exploring the Concept of Moral Distress with Community-Based Researchers: An Australian Study,” *Journal of Social Service Research* 37, no. 1 (2011): 73-85; Showalter, B. L., et al., “Moral Distress in Clinical Research Nurses,” *Nursing Ethics* 29 (2022): 1697-1708. 27795869 10.1080/21507716.2013.806969 PMC5082423

[4] Epstein, E. G. , et al., “Enhancing Understanding of Moral Distress: The Measure of Moral Distress for Health Care Professionals,” AJOB Empirical Bioethics 10, no. 2 (2019): 113–24. 31002584 10.1080/23294515.2019.1586008

[5] Sandelowski, M. , “Whatever Happened to Qualitative Description?,” Research in Nursing & Health 23, no. 4 (2000): 334–40, at 336. 10940958 10.1002/1098-240x(200008)23:4<334::aid-nur9>3.0.co;2-g

[6] Ibid.

[7] Leavy, P. , Research Design: Quantitative, Qualitative, Mixed Methods, Arts-Based, and Community-Based Participatory Research Approaches (New York: Guilford Press, 2017)

[8] Bowen, G. A. , “Grounded Theory and Sensitizing Concepts,” International Journal of Qualitative Methods 5, no. 3 (2006): 12–23.

[9] McCarthy, J. , and R. Deady , “Moral Distress Reconsidered,” Nursing Ethics 15, no. 2 (2008): 254–62; Hanna, D. R., “Moral Distress: The State of the Science,” *Research and Theory for Nursing Practice* 18, no. 1 (2004): 73-93; Hamric, A. B., “Empirical Research on Moral Distress: Issues, Challenges, and Opportunities,” *HEC Forum* 24, no. 1 (2012): 39-49.18272615

[10] Fortune Business Insights , Clinical Trials Market Size, Share & COVID-19 Impact Analysis, by Phase (Phase I, Phase II, Phase III, and Phase IV), by Application (Oncology, CNS Disorder, Cardiology, Infectious Disease, Metabolic Disorder, Renal/Nephrology, and Others), and Regional Forecast, 2023-2030 (Fortune Business Insights, October 28, 2024), https://www.fortunebusinessinsights.com/clinical-trials-market-106930.

[11] Tinkler, L. , et al., “Professional Identity and the Clinical Research Nurse: A Qualitative Study Exploring Issues Having an Impact on Participant Recruitment in Research,” Journal of Advanced Nursing 74, no. 2 (2018): 318–28, at 326. 28792610 10.1111/jan.13409

[12] Ibid.

[13] Jameton, A. , “What Moral Distress in Nursing History Could Suggest about the Future of Health Care,” AMA Journal of Ethics 19 (2017): 617–28. 28644792 10.1001/journalofethics.2017.19.6.mhst1-1706

[14] Prompahakul, C. , and E. G. Epstein , “Moral Distress Experienced by Non-Western Nurses: An Integrative Review,” Nursing Ethics 27 (2020): 778–95; Moses, L., et al., “Ethical Conflict and Moral Distress in Veterinary Practice: A Survey of North American Veterinarians,” *Journal of Veterinary Internal Medicine* 32 (2018): 2115-22; Prentice, T., et al., “Moral Distress within Neonatal and Paediatric Intensive Care Units: A Systematic Review,” *Archives of Disease in Childhood* 101 (2016): 701-8.31750780

[15] Larkin, M. E. , et al., “Ethical Challenges Experienced by Clinical Research Nurses: A Qualitative Study,” Nursing Ethics 26, no. 1 (2019): 172–84. 29281934 10.1177/0969733017693441

[16] Ibid., 179.

[17] Davis, A. M. , et al . “The Invisible Hand in Clinical Research: The Study Coordinator's Critical Role in Human Subjects Protection,” Journal of Law, Medicine, & Ethics 30, no. 3 (2002): 411–19 (1-19 in online version). 10.1111/j.1748-720x.2002.tb00410.x PMC4819323 12497701

[18] Ibid., 7.

[19] Ibid., 4.

[20] Sunderland et al., “Exploring the Concept of Moral Distress with Community-Based Researchers.”

[21] Prompahakul C. , et al., “Moral Distress Among Nurses: A Mixed-Methods Study,” Nursing Ethics 28 (2021): 1165–82; Prompahakul and Epstein, “Moral Distress Experienced by Non-Western Nurses”; Trautmann J., et al., “Relationships Among Moral Distress, Level of Practice Independence, and Intent to Leave of Nurse Practitioners in Emergency Departments: Results from a National Survey,” *Advanced Emergency Nursing Journal* 37, no. 3 (2015): 134-45. 25929224 10.1097/TME.0000000000000060

[22] Davis et al. “The Invisible Hand in Clinical Research.”

[23] Brunnquell, D. , and C. Michaelson , “Moral Hazard in Pediatrics,” American Journal of Bioethics 16, no. 7 (2016): 29–38; Marshall, M. F., and E. G. Epstein, “Moral Hazard and Moral Distress: A Marriage Made in Purgatory,” *American Journal of Bioethics* 16, no. 7 (2016): 46-48. 10.1080/15265161.2016.1180441 27292845

[24] Lamiani, G. , L. Borghi , and P. Argentero , “When Healthcare Professionals Cannot Do the Right Thing: A Systematic Review of Moral Distress and Its Correlates,” Journal of Health Psychology 22, no. 1 (2017): 51–67; Showalter et al., “Moral Distress in Clinical Research Nurses”; Fisher et al., “Moral Stress, Moral Practice, and Ethical Climate in Community-Based Drug-Use Research.” 26220460 10.1177/1359105315595120

[25] Ibid., at 28.

[26] Deschenes, S. , et al., “Moral Distress: A Concept Clarification,” Nursing Ethics 27 (2020): 1127–46; Epstein, E. G., and A. R. Hurst, “Looking at the Positive Side of Moral Distress: Why It's a Problem,” *Journal of Clinical Ethics* 28, no. 1 (2017): 37-41; Austin, W., “Moral Distress and the Contemporary Plight of Health Professionals,” *HEC Forum* 24, no. 1 (2012): 27-38. 22441996 10.1007/s10730-012-9179-8

[27] Epstein and Hurst , “Looking at the Positive Side of Moral Distress.” 28436927

[28] Sharp, R. R. , et al., “Research Ethics Consultation: Ethical and Professional Practice Challenges and Recommendations,” Academic Medicine 90, no. 5 (2015): 615–20, at 615. 25607942 10.1097/ACM.0000000000000640 PMC4414686

[29] McCormick, J. B. , et al., “The Establishment of Research Ethics Consultation Services (RECS): An Emerging Research Resource,” Clinical and Translational Science 6, no. 1 (2013): 40–44. 23399088 10.1111/cts.12008 PMC3573851

[30] Hamric, A. B. , and E. G. Epstein , “A Health System-Wide Moral Distress Consultation Service: Development and Evaluation,” HEC Forum 29, no. 2 (2017): 127–43. 28070806 10.1007/s10730-016-9315-y

[31] Canadian Centre of Excellence on PTSD and the Phoenix Australia Centre for Posttraumatic Mental Health , Racial Inequities and Moral Distress: A Supplement to Moral Stress Amongst Healthcare Workers during COVID-19: A Guide to Moral Injury, Canadian Centre of Excellence on PTSD and the Phoenix Australia Centre for Posttraumatic Mental Health, accessed May 6, 2022, https://www.moralinjuryguide.ca/Documents/racial-inequities-and-moral-distress.pdf, pp. 1–18, at 3.

[32] Epstein et al., “Moral Distress, Mattering, and Secondary Traumatic Stress in Provider Burnout,” 149. 10.4037/aacnacc2020285 32525997

[33] Burnett-Bowie, S. M. , et al., “Attitudes and Actions Related to Racism: The Anti-RaCism (ARC) Survey Study,” Journal of General Internal Medicine 37 (2022): 2337–44; Florez, M. I., et al., “Improving Diversity in Clinical Trial Volunteer Participation by Addressing Racial and Ethnic Representation Among the Clinical Research Workforce,” *Applied Clinical Trials* (2022), https://www.appliedclinicaltrialsonline.com/view/improving-diversity-in-clinical-trial-volunteer-participation-by-addressing-racial-and-ethnic-representation-among-the-clinical-research-workforce. 35157198 10.1007/s11606-021-07385-1 PMC9360374

[34] Wocial, L. D. , and M. T. Weaver , “Development and Psychometric Testing of a New Tool for Detecting Moral Distress: The Moral Distress Thermometer,” Journal of Advanced Nursing 69, no. 1 (2013): 167–74; Epstein et al., “Enhancing Understanding of Moral Distress.” 22607094 10.1111/j.1365-2648.2012.06036.x

