# Supplementary material for: Investigating Moral Distress in Clinical Research Professionals—A Deep Dive into Troubled Waters
Source: Ethics Hum Res. 2025 Jan 3;47(1):34–45. doi: 10.1002/eahr.60006 (PMC11696204; doi:10.1002/eahr.60006)
Supplement: Supplementary file 1 — Supporting information [file EAHR-47-34-s001.pdf]

Table 2. Themes and Subthemes with Illustrative Quotations

Theme	Subtheme	Illustrative quote(s)
Commodification of research	Financial incentives having undue influence over decision-making	"I think it was the grants. Just to find 'Will we get funding from this sponsor again?' and it's like 'If we don't meet our targets, we're in jeopardy of not getting renewal.' And we got the renewal, but it was very tenuous and I think everyone was nervous it was going to get canceled. So there was a lot of pressure to produce some results. We were really early in the [Covid-19] pandemic, I just don't know that we can do that. It was definitely grant-related, I think." (I.4)
	Pressure to enroll subjects ("making subjects fit" into studies)	"But it was these little things that in [the PI's] mind were not determined to be a safety issue with the patient, even though it was going against the protocol itself. Like, 'How many [episodes] do you have?' / 'Well I'm sure they have more [episodes] than that; they're fine for the study.' So it was things like that, and it was just so clear that it was all finance for [the PI]. It wasn't research to bring about change, or to help people, or to do your part." (I.3)
Concern for subjects	Requirements of study conflicting with needs of subject	"...it's not required to be in the protocol. I don't even offer it to people with questionable kidneys because I don't want to kill their kidneys with this [test]. But [is my colleague] doing a better job of getting the [clinical research] company their data? Yeah, because most of [my colleague's] people get [tests] but none of my people get [tests]." (I.1)<br><br>"I did see a patient recently who our team thought 'Maybe she's not the best candidate' but we pushed her through and she got [research treatment]. She is doing okay, but she's pretty weak and she's [elderly], and I couldn't help wondering if she got treated versus her not being treated, how her quality of life would have been. But when I saw her, she looked

		pretty weak, and so I felt a little sad for her." (I.10)
	Compromised informed consent	"So it's hard to tell, is this really truly an informed consent, where you're really trying to make a good decision, with all the risks and the benefits, or are you feeling like 'Well this fancy doctor that my local doctor told me about thinks it's a good idea [to participate in the study] and I don't have any other options so there's no choice, I have to do this if I want to feel better.'" (I.1)
	Blurred boundaries between research and care	"So I think sometimes the patients could worry that if they said no, that the [PI] wouldn't want to perform the operation on them, or they might not do it as well, so I could see how the boundaries would blur a little bit when it's like 'Am I in a research study or am I still here to get my procedure?'" (I.2)
	Attention to subject vulnerability	"Just having that piece too made me wonder, if [the research subject] had a stronger family support system, if that would have even made a difference too. Because it was just him. And I don't know how much of it he really wanted to be involved in, during the course [of the trial]. Of course, he signed consent for the clinical trial and so forth, but I don't know, as the trial went on, if he really understood everything that was involved and if he really wanted to participate." (I.9)
	Inadequate compensation	*Insufficient*: "You can't just ask them questions, give them nothing in return, and use their data. That's so messed up. I felt it was super unethical." (I.4)<br><br>*Excessive*: " In one of my studies it's an [operation] and the [device] itself is, right now, like [tens of thousands of dollars] – just the actual [device], not the procedure, not the hospital stay, none of that. But if you do the

		study, it would be free. If you have crummy insurance or you don't have insurance, it's kind of like you've got them over a barrel. They're going to say yes." (I.1)
Compromised science	Questionable data reliability	"...a lot of times it made me kind of question the integrity of the research data that was coming in, because if you're just changing so many things last minute it's almost like you're fishing for the answers that you want to find." (I.2)
	Unreliable/incompetent research team	"You need to check behind regulatory, because regulatory doesn't know regulatory. So you need to follow up and make sure these things are done…So it was constantly double checking. I felt like the main part of my job was double checking. Double checking regulatory. Double checking the visits to make sure that [the PI] did everything that [they] were supposed to do. And that is major, when you don't trust that others are able to do their jobs, and it falls to you to make sure that these things are done correctly." (I.3)
	Insufficient oversight	"I really, really have a hard time finding the right person to contact to get my question answered. It can be a good period of time until I get someone on the phone for an issue that's happening immediately with the patient." (I.5)
	Insufficient training	"A lot of it is left up to [CRPs] just kind of trying to figure things out on our own. And you're lucky if you have a supervisor who will put in the time or try to put in the time to train you on certain things, but a lot of the time, we're not getting the support that we need. And so that's a segue into another thing morally that's been challenging over time in working in the research field is lack of training and support. It's not consistent." (I.6)
Structures of hierarchy	Uncooperative study leadership	"I was clearly not in the position to make those remarks. The worst part for me was that my PI

		[is] the one that came up with a study…[They] were not at all receptive to any criticism or feedback." (I.4)
	Inflexibility of study design	"It was just very frustrating to me because I was supposed to be advising on that, and I'm like 'I feel like you're forcing me to ignore what the people on the ground [the study participants] are saying.' And they're all saying 'What are you doing with this research; why are you doing this now?' And part of it was our grant deadline: they wanted certain targets by certain times, and I'm like 'This is just not possible.' And [the study leadership is] not listening to what the research participants are saying, and as a result, we had terrible uptake." (I.4)
	Feelings of powerlessness	"So sometimes there's disagreement between what I might feel is appropriate and best for the patient and what my PI is suggesting. And, ultimately, because I work for the PI – and [my PI] is a physician and [is] very experienced – we can't just blatantly say 'No, I don't agree with you.' Well, I guess we could, but it's uncomfortable to push back on that sometimes." (I.10)
	Fear of retribution	"We all needed our jobs. Needed to pay my mortgage. Needed to feed my dog. Needed to feed myself. And that just put us in a really bad position of always being on edge. Not knowing what would happen that would come back on us, that we did something when we knew that we didn't." (I.3)
Responses to moral distress	Self-mitigation	"You just know at a certain point no one's going to do anything, even if you go barking off. So you just have to do what you have to do to make sure things are covered. So from that moment on, I decided I would do – as best I could [given my professional background] – my own exam of the patient before [the PI] did… I wanted to make sure [the PI] wasn't missing something

		even though [they] were signing off saying [they] did the full physical exam. I don't know how it's possible in a minute, but [they] said it was. So that helped me to be more assertive without being aggressive, because [the PI] would not have taken that well." (I.5)
	Solidarity	"Essentially, myself and the immediate supervisor and the rest of the [CRP] team were like 'Let's talk together about when issues do come up before we present them to the PI to see if we can work through them without getting [the PI] involved, because we don't want to continue having more retention issues.' So we had a workaround in a way, but of course that's never the route you want to go with projects." (I.6)
	Deferral to higher-ups	"Personally, I think [my PI is] very professional and an excellent doctor. I wouldn't necessarily say [they're] the most approachable… But if [they] told me to do something, I would defer to [them]. I would just do what [they] say, and just absorb whatever negative feeling I might have." (I.10)
	Avoidance	*Emotional* - "I guess for myself, I have to put on my research hat and just think 'This will help down the road' and try not to let the emotion side or 'What if I was that patient?' – I try not to do too much of that." (I.9)<br><br>*Interpersonal* - "Those were definitely times where I felt really uneasy about what was going on. It was just a lot. Over time, if I knew that I could avoid certain doctors – in terms of them screening the patient for an enrollment like that – then I would." (I.6)
	Quitting	"And a project we worked on had a lot of focus on [clinical metric], and I did not think that it was appropriate for the patient population we were working with, and that is one of the big

		reasons why I decided that I needed to look for a different position, because I had such an internal conflict working on that study." (I.6)<br><br>"That was definitely one of the reasons why I left this project and decided to [leave clinical research]. I was just like, 'This is not going to work, long-term.'" (I.4)
	Speaking out	*Positive impact* - "I one day was like 'I don't think you [the PI] should talk to people about the studies in any sort of detail. I think you should say, 'Are you interested in hearing about research options?' and you can introduce them really broadly and then I can go into detail alone in the room with the patient so that they feel more comfortable saying no'…I think that kind of got better when I did talk to [my PI]." (I.1)<br><br>*Negative impact* - "I went again and again and again, and had meetings like 'This person is not working out. They're under me, I'm supposed to be training them. They do not want to be trained, they are sure that they know what they're doing'…And I was told that I was not capable of teaching this person. And that I needed to do better." (I.3)
